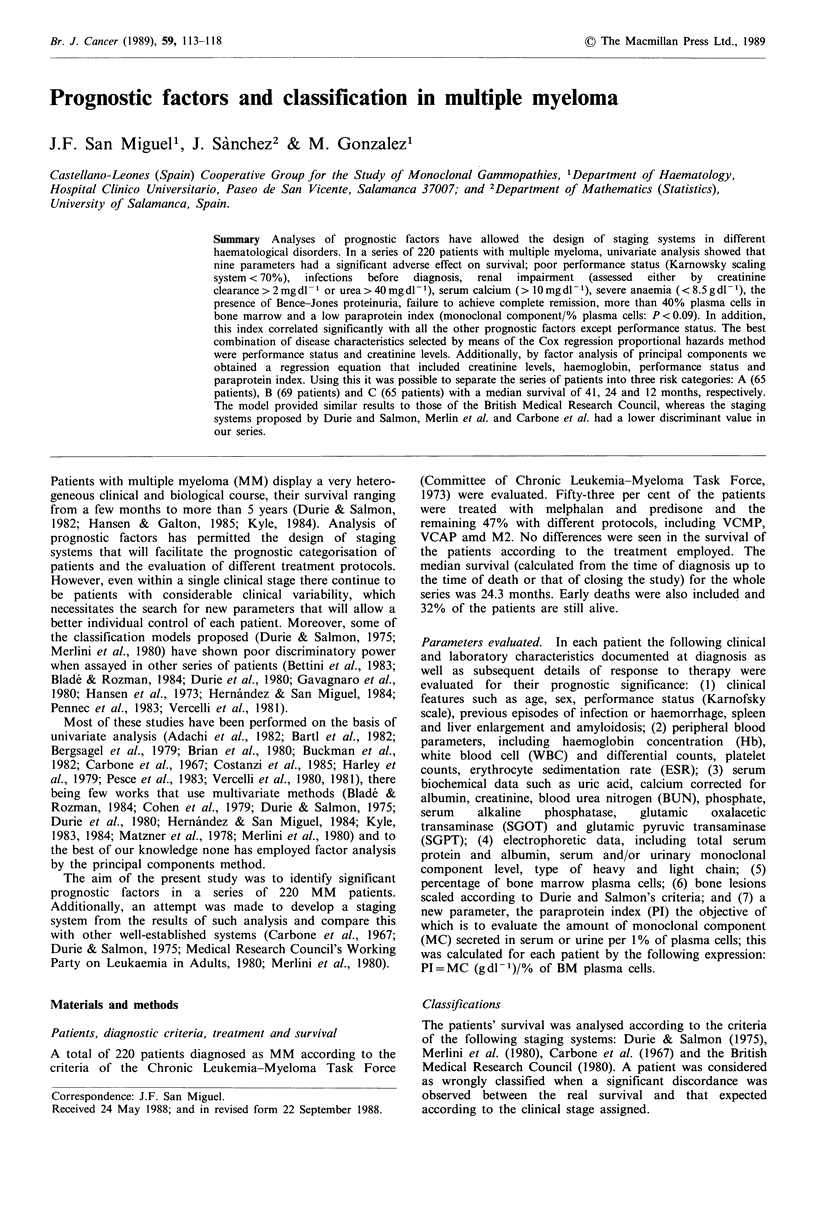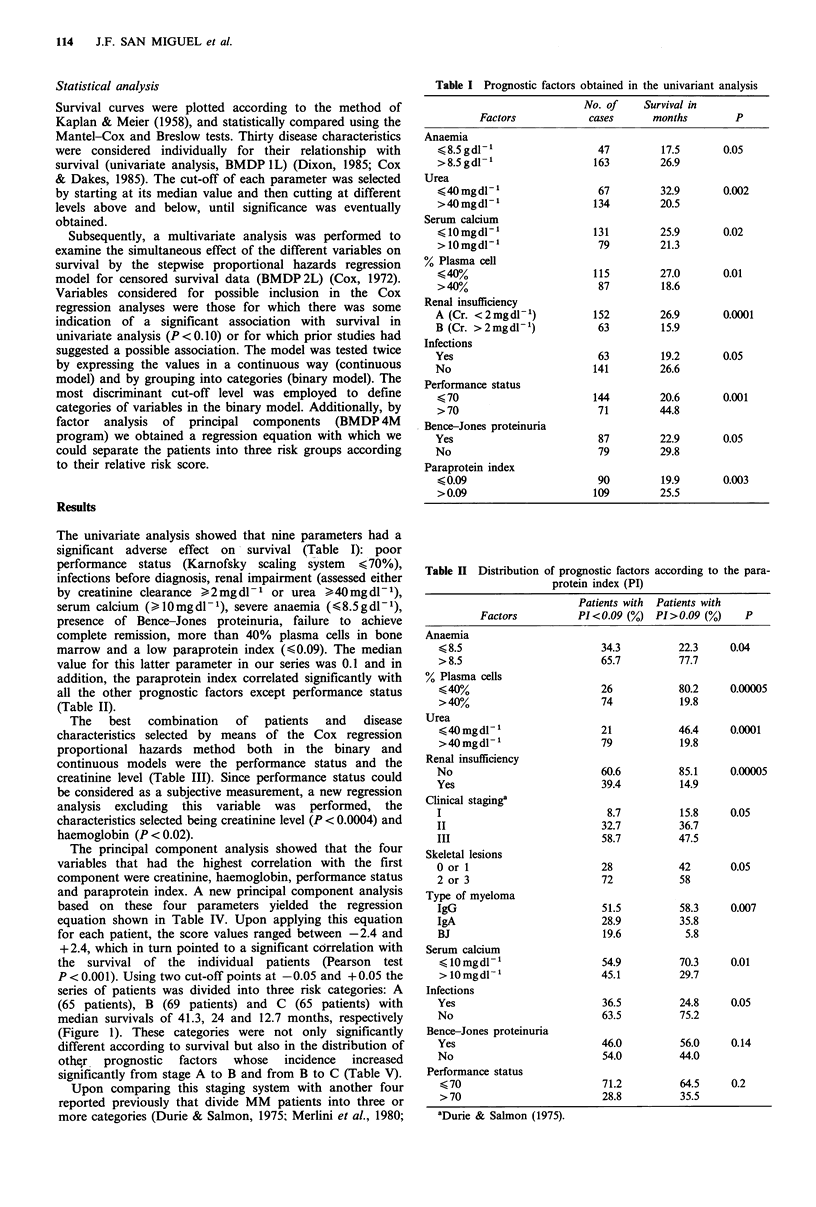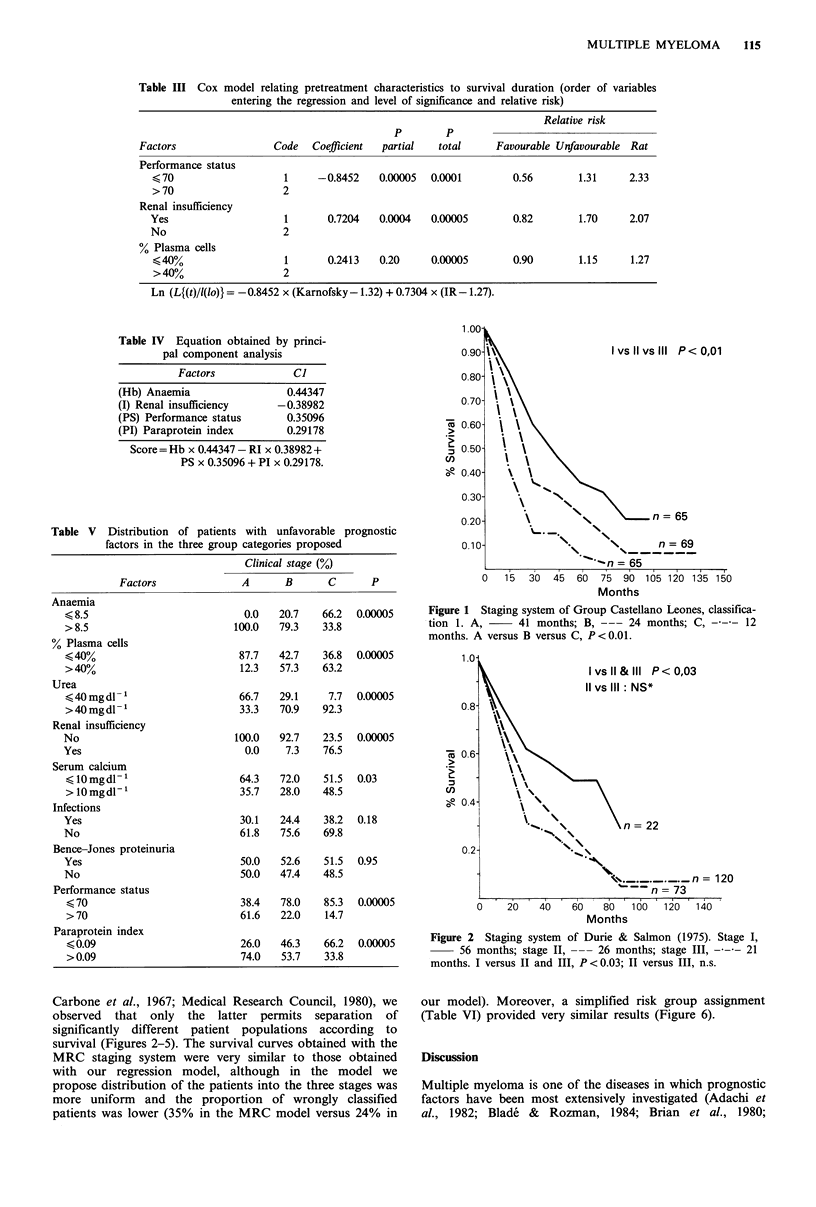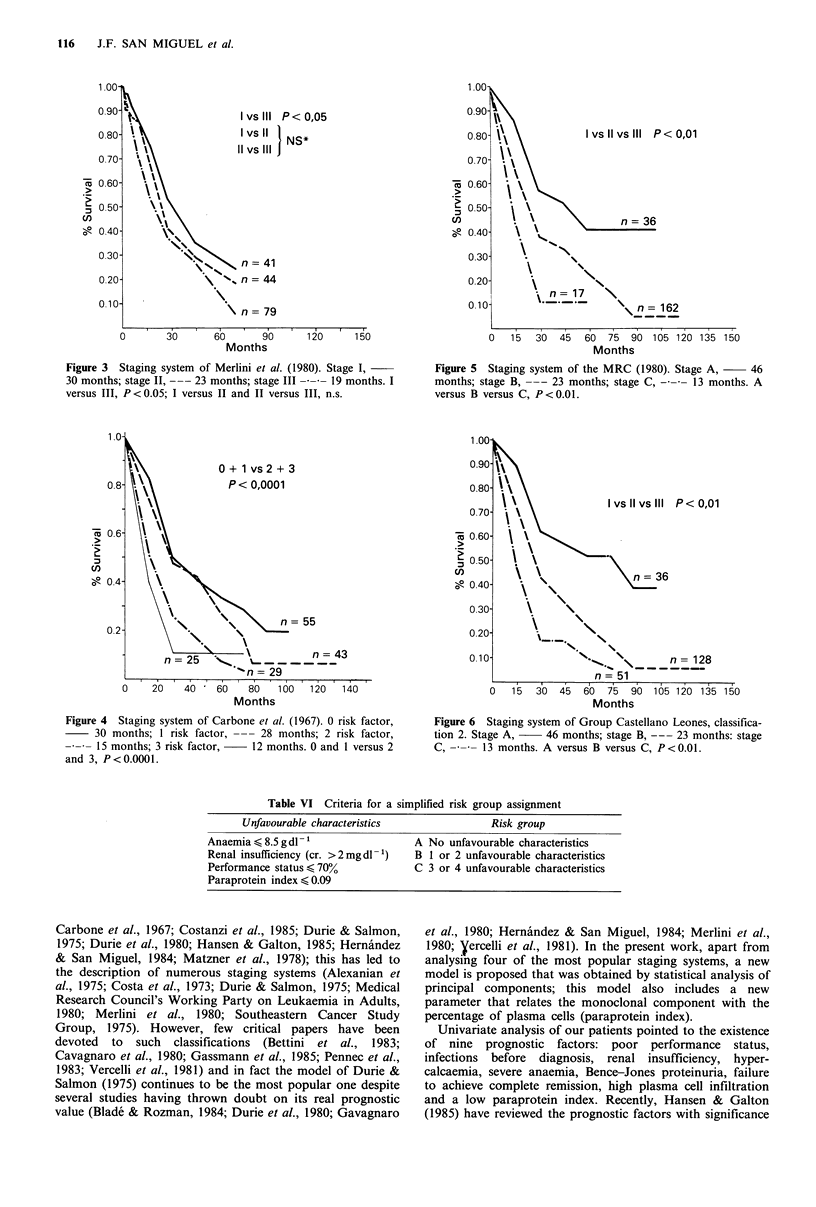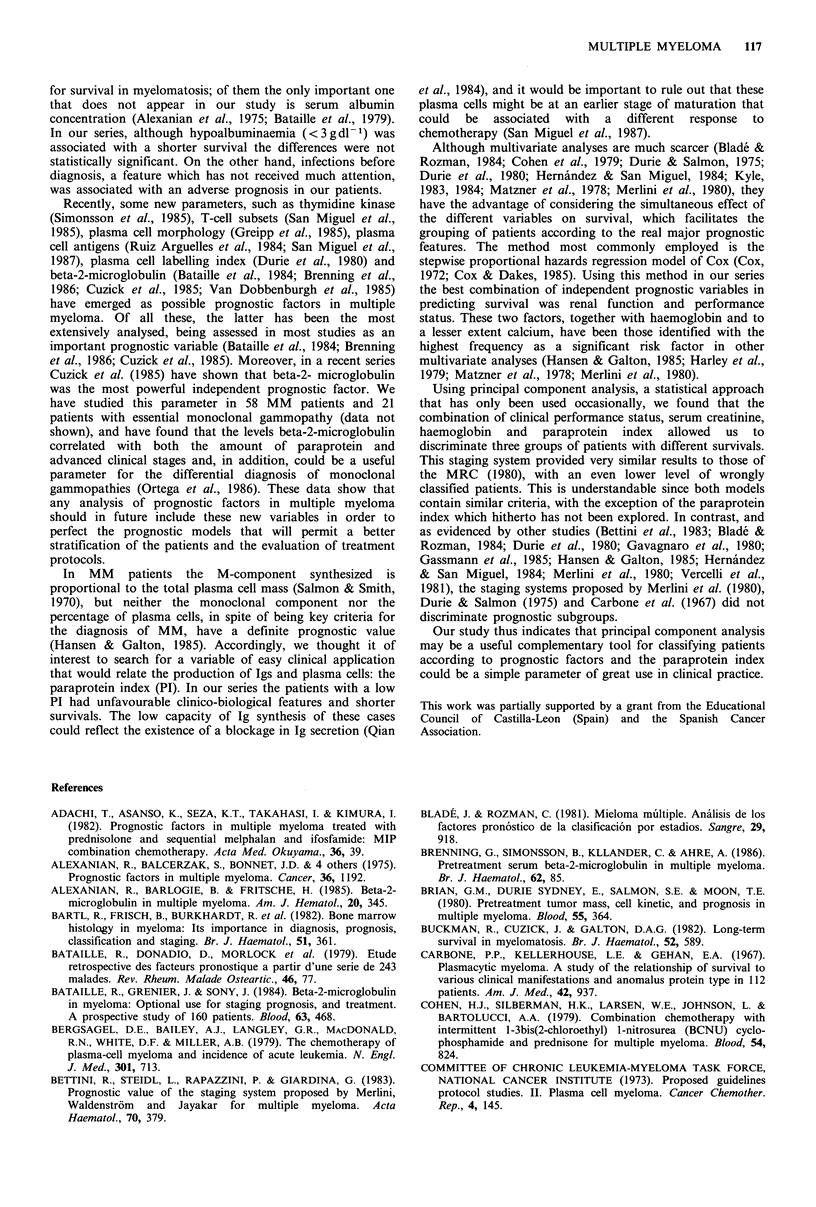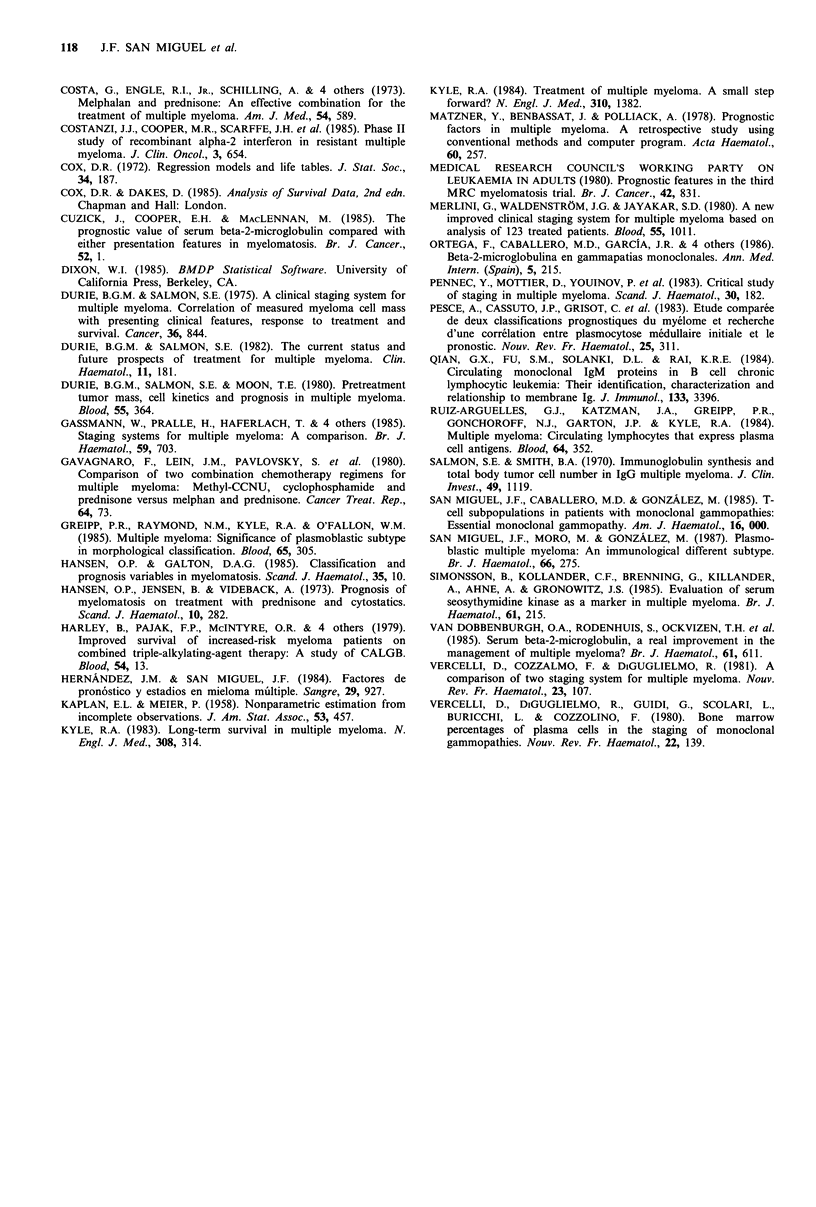# Prognostic factors and classification in multiple myeloma.

**DOI:** 10.1038/bjc.1989.23

**Published:** 1989-01

**Authors:** J. F. San Miguel, J. SÃ nchez, M. Gonzalez

**Affiliations:** Department of Haematology, Hospital Clinico Universitario, Salamanca, Spain.

## Abstract

Analyses of prognostic factors have allowed the design of staging systems in different haematological disorders. In a series of 220 patients with multiple myeloma, univariate analysis showed that nine parameters had a significant adverse effect on survival; poor performance status (Karnowsky scaling system less than 70%), infections before diagnosis, renal impairment (assessed either by creatinine clearance greater than 2 mg dl-1 or urea greater than 40 mg dl-1), serum calcium (greater than 10 mg dl-1), severe anaemia (less than 8.5 g dl-1), the presence of Bence-Jones proteinuria, failure to achieve complete remission, more than 40% plasma cells in bone marrow and a low paraprotein index (monoclonal component/% plasma cells: P less than 0.09). In addition, this index correlated significantly with all the other prognostic factors except performance status. The best combination of disease characteristics selected by means of the Cox regression proportional hazards method were performance status and creatinine levels. Additionally, by factor analysis of principal components we obtained a regression equation that included creatinine levels, haemoglobin, performance status and paraprotein index. Using this it was possible to separate the series of patients into three risk categories: A (65 patients), B (69 patients) and C (65 patients) with a median survival of 41, 24 and 12 months, respectively. The model provided similar results to those of the British Medical Research Council, whereas the staging systems proposed by Durie and Salmon, Merlin et al. and Carbone et al. had a lower discriminant value in our series.


Br. J. Cancer (1989), 59, 113–118 © The Macmillan Press Ltd., 1989

### Prognostic factors and classification in multiple myeloma

J.F. San Miguel[1], J. Sànchez[2] & M. Gonzalez[1]

*Castellano-Leones (Spain) Cooperative Group for the Study of Monoclonal Gammopathies, [1]Department of Haematology, Hospital Clinico Universitario, Paseo de San Vicente, Salamanca 37007; and [2]Department of Mathematics (Statistics), University of Salamanca, Spain.*

**Summary** Analyses of prognostic factors have allowed the design of staging systems in different haematological disorders. In a series of 220 patients with multiple myeloma, univariate analysis showed that nine parameters had a significant adverse effect on survival; poor performance status (Karnowsky scaling system < 70%), infections before diagnosis, renal impairment (assessed either by creatinine clearance > 2 mg dl$^{-1}$ or urea > 40 mg dl$^{-1}$), serum calcium (> 10 mg dl$^{-1}$), severe anaemia (< 8.5 g dl$^{-1}$), the presence of Bence–Jones proteinuria, failure to achieve complete remission, more than 40% plasma cells in bone marrow and a low paraprotein index (monoclonal component/% plasma cells: $P < 0.09$). In addition, this index correlated significantly with all the other prognostic factors except performance status. The best combination of disease characteristics selected by means of the Cox regression proportional hazards method were performance status and creatinine levels. Additionally, by factor analysis of principal components we obtained a regression equation that included creatinine levels, haemoglobin, performance status and paraprotein index. Using this it was possible to separate the series of patients into three risk categories: A (65 patients), B (69 patients) and C (65 patients) with a median survival of 41, 24 and 12 months, respectively. The model provided similar results to those of the British Medical Research Council, whereas the staging systems proposed by Durie and Salmon, Merlin *et al.* and Carbone *et al.* had a lower discriminant value in our series.

Patients with multiple myeloma (MM) display a very heterogeneous clinical and biological course, their survival ranging from a few months to more than 5 years (Durie & Salmon, 1982; Hansen & Galton, 1985; Kyle, 1984). Analysis of prognostic factors has permitted the design of staging systems that will facilitate the prognostic categorisation of patients and the evaluation of different treatment protocols. However, even within a single clinical stage there continue to be patients with considerable clinical variability, which necessitates the search for new parameters that will allow a better individual control of each patient. Moreover, some of the classification models proposed (Durie & Salmon, 1975; Merlini *et al.*, 1980) have shown poor discriminatory power when assayed in other series of patients (Bettini *et al.*, 1983; Bladé & Rozman, 1984; Durie *et al.*, 1980; Gavagnaro *et al.*, 1980; Hansen *et al.*, 1973; Hernández & San Miguel, 1984; Pennec *et al.*, 1983; Vercelli *et al.*, 1981).

Most of these studies have been performed on the basis of univariate analysis (Adachi *et al.*, 1982; Bartl *et al.*, 1982; Bergsagel *et al.*, 1979; Brian *et al.*, 1980; Buckman *et al.*, 1982; Carbone *et al.*, 1967; Costanzi *et al.*, 1985; Harley *et al.*, 1979; Pesce *et al.*, 1983; Vercelli *et al.*, 1980, 1981), there being few works that use multivariate methods (Bladé & Rozman, 1984; Cohen *et al.*, 1979; Durie & Salmon, 1975; Durie *et al.*, 1980; Hernández & San Miguel, 1984; Kyle, 1983, 1984; Matzner *et al.*, 1978; Merlini *et al.*, 1980) and to the best of our knowledge none has employed factor analysis by the principal components method.

The aim of the present study was to identify significant prognostic factors in a series of 220 MM patients. Additionally, an attempt was made to develop a staging system from the results of such analysis and compare this with other well-established systems (Carbone *et al.*, 1967; Durie & Salmon, 1975; Medical Research Council's Working Party on Leukaemia in Adults, 1980; Merlini *et al.*, 1980).

### Materials and methods

### *Patients, diagnostic criteria, treatment and survival*

A total of 220 patients diagnosed as MM according to the criteria of the Chronic Leukemia–Myeloma Task Force (Committee of Chronic Leukemia–Myeloma Task Force, 1973) were evaluated. Fifty-three per cent of the patients were treated with melphalan and predisone and the remaining 47% with different protocols, including VCMP, VCAP amd M2. No differences were seen in the survival of the patients according to the treatment employed. The median survival (calculated from the time of diagnosis up to the time of death or that of closing the study) for the whole series was 24.3 months. Early deaths were also included and 32% of the patients are still alive.

*Parameters evaluated.* In each patient the following clinical and laboratory characteristics documented at diagnosis as well as subsequent details of response to therapy were evaluated for their prognostic significance: (1) clinical features such as age, sex, performance status (Karnofsky scale), previous episodes of infection or haemorrhage, spleen and liver enlargement and amyloidosis; (2) peripheral blood parameters, including haemoglobin concentration (Hb), white blood cell (WBC) and differential counts, platelet counts, erythrocyte sedimentation rate (ESR); (3) serum biochemical data such as uric acid, calcium corrected for albumin, creatinine, blood urea nitrogen (BUN), phosphate, serum alkaline phosphatase, glutamic oxalacetic transaminase (SGOT) and glutamic pyruvic transaminase (SGPT); (4) electrophoretic data, including total serum protein and albumin, serum and/or urinary monoclonal component level, type of heavy and light chain; (5) percentage of bone marrow plasma cells; (6) bone lesions scaled according to Durie and Salmon's criteria; and (7) a new parameter, the paraprotein index (PI) the objective of which is to evaluate the amount of monoclonal component (MC) secreted in serum or urine per 1% of plasma cells; this was calculated for each patient by the following expression: PI = MC (g dl$^{-1}$)/% of BM plasma cells.

### *Classifications*

The patients' survival was analysed according to the criteria of the following staging systems: Durie & Salmon (1975), Merlini *et al.* (1980), Carbone *et al.* (1967) and the British Medical Research Council (1980). A patient was considered as wrongly classified when a significant discordance was observed between the real survival and that expected according to the clinical stage assigned.

Correspondence: J.F. San Miguel.
Received 24 May 1988; and in revised form 22 September 1988.

*Statistical analysis*

Survival curves were plotted according to the method of Kaplan & Meier (1958), and statistically compared using the Mantel–Cox and Breslow tests. Thirty disease characteristics were considered individually for their relationship with survival (univariate analysis, BMDP 1L) (Dixon, 1985; Cox & Dakes, 1985). The cut-off of each parameter was selected by starting at its median value and then cutting at different levels above and below, until significance was eventually obtained.

Subsequently, a multivariate analysis was performed to examine the simultaneous effect of the different variables on survival by the stepwise proportional hazards regression model for censored survival data (BMDP 2L) (Cox, 1972). Variables considered for possible inclusion in the Cox regression analyses were those for which there was some indication of a significant association with survival in univariate analysis ($P < 0.10$) or for which prior studies had suggested a possible association. The model was tested twice by expressing the values in a continuous way (continuous model) and by grouping into categories (binary model). The most discriminant cut-off level was employed to define categories of variables in the binary model. Additionally, by factor analysis of principal components (BMDP 4M program) we obtained a regression equation with which we could separate the patients into three risk groups according to their relative risk score.

### Results

The univariate analysis showed that nine parameters had a significant adverse effect on survival (Table I): poor performance status (Karnofsky scaling system ≤70%), infections before diagnosis, renal impairment (assessed either by creatinine clearance ≥2 mg dl$^{-1}$ or urea ≥40 mg dl$^{-1}$), serum calcium (≥10 mg dl$^{-1}$), severe anaemia (≤8.5 g dl$^{-1}$), presence of Bence–Jones proteinuria, failure to achieve complete remission, more than 40% plasma cells in bone marrow and a low paraprotein index (≤0.09). The median value for this latter parameter in our series was 0.1 and in addition, the paraprotein index correlated significantly with all the other prognostic factors except performance status (Table II).

The best combination of patients and disease characteristics selected by means of the Cox regression proportional hazards method both in the binary and continuous models were the performance status and the creatinine level (Table III). Since performance status could be considered as a subjective measurement, a new regression analysis excluding this variable was performed, the characteristics selected being creatinine level ($P < 0.0004$) and haemoglobin ($P < 0.02$).

The principal component analysis showed that the four variables that had the highest correlation with the first component were creatinine, haemoglobin, performance status and paraprotein index. A new principal component analysis based on these four parameters yielded the regression equation shown in Table IV. Upon applying this equation for each patient, the score values ranged between −2.4 and +2.4, which in turn pointed to a significant correlation with the survival of the individual patients (Pearson test $P < 0.001$). Using two cut-off points at −0.05 and +0.05 the series of patients was divided into three risk categories: A (65 patients), B (69 patients) and C (65 patients) with median survivals of 41.3, 24 and 12.7 months, respectively (Figure 1). These categories were not only significantly different according to survival but also in the distribution of other prognostic factors whose incidence increased significantly from stage A to B and from B to C (Table V).

Upon comparing this staging system with another four reported previously that divide MM patients into three or more categories (Durie & Salmon, 1975; Merlini et al., 1980;

**Table I** Prognostic factors obtained in the univariant analysis

| *Factors* | *No. of cases* | *Survival in months* | *P* |
|---|---|---|---|
| Anaemia | | | |
| ≤8.5 g dl$^{-1}$ | 47 | 17.5 | 0.05 |
| >8.5 g dl$^{-1}$ | 163 | 26.9 | |
| Urea | | | |
| ≤40 mg dl$^{-1}$ | 67 | 32.9 | 0.002 |
| >40 mg dl$^{-1}$ | 134 | 20.5 | |
| Serum calcium | | | |
| ≤10 mg dl$^{-1}$ | 131 | 25.9 | 0.02 |
| >10 mg dl$^{-1}$ | 79 | 21.3 | |
| % Plasma cell | | | |
| ≤40% | 115 | 27.0 | 0.01 |
| >40% | 87 | 18.6 | |
| Renal insufficiency | | | |
| A (Cr. <2 mg dl$^{-1}$) | 152 | 26.9 | 0.0001 |
| B (Cr. >2 mg dl$^{-1}$) | 63 | 15.9 | |
| Infections | | | |
| Yes | 63 | 19.2 | 0.05 |
| No | 141 | 26.6 | |
| Performance status | | | |
| ≤70 | 144 | 20.6 | 0.001 |
| >70 | 71 | 44.8 | |
| Bence–Jones proteinuria | | | |
| Yes | 87 | 22.9 | 0.05 |
| No | 79 | 29.8 | |
| Paraprotein index | | | |
| ≤0.09 | 90 | 19.9 | 0.003 |
| >0.09 | 109 | 25.5 | |

**Table II** Distribution of prognostic factors according to the paraprotein index (PI)

| *Factors* | *Patients with PI<0.09 (%)* | *Patients with PI>0.09 (%)* | *P* |
|---|---|---|---|
| Anaemia | | | |
| ≤8.5 | 34.3 | 22.3 | 0.04 |
| >8.5 | 65.7 | 77.7 | |
| % Plasma cells | | | |
| ≤40% | 26 | 80.2 | 0.00005 |
| >40% | 74 | 19.8 | |
| Urea | | | |
| ≤40 mg dl$^{-1}$ | 21 | 46.4 | 0.0001 |
| >40 mg dl$^{-1}$ | 79 | 19.8 | |
| Renal insufficiency | | | |
| No | 60.6 | 85.1 | 0.00005 |
| Yes | 39.4 | 14.9 | |
| Clinical staging[a] | | | |
| I | 8.7 | 15.8 | 0.05 |
| II | 32.7 | 36.7 | |
| III | 58.7 | 47.5 | |
| Skeletal lesions | | | |
| 0 or 1 | 28 | 42 | 0.05 |
| 2 or 3 | 72 | 58 | |
| Type of myeloma | | | |
| IgG | 51.5 | 58.3 | 0.007 |
| IgA | 28.9 | 35.8 | |
| BJ | 19.6 | 5.8 | |
| Serum calcium | | | |
| ≤10 mg dl$^{-1}$ | 54.9 | 70.3 | 0.01 |
| >10 mg dl$^{-1}$ | 45.1 | 29.7 | |
| Infections | | | |
| Yes | 36.5 | 24.8 | 0.05 |
| No | 63.5 | 75.2 | |
| Bence–Jones proteinuria | | | |
| Yes | 46.0 | 56.0 | 0.14 |
| No | 54.0 | 44.0 | |
| Performance status | | | |
| ≤70 | 71.2 | 64.5 | 0.2 |
| >70 | 28.8 | 35.5 | |

[a]Durie & Salmon (1975).

**Table III** Cox model relating pretreatment characteristics to survival duration (order of variables entering the regression and level of significance and relative risk)

| *Factors* | *Code* | *Coefficient* | *P partial* | *P total* | *Relative risk* Favourable | Unfavourable | Rat |
|---|---|---|---|---|---|---|---|
| Performance status | | | | | | | |
| ≤70 | 1 | −0.8452 | 0.00005 | 0.0001 | 0.56 | 1.31 | 2.33 |
| >70 | 2 | | | | | | |
| Renal insufficiency | | | | | | | |
| Yes | 1 | 0.7204 | 0.0004 | 0.00005 | 0.82 | 1.70 | 2.07 |
| No | 2 | | | | | | |
| % Plasma cells | | | | | | | |
| ≤40% | 1 | 0.2413 | 0.20 | 0.00005 | 0.90 | 1.15 | 1.27 |
| >40% | 2 | | | | | | |

$\mathrm{Ln}\ (L\{(t)/l(lo)\} = -0.8452 \times (\mathrm{Karnofsky} - 1.32) + 0.7304 \times (\mathrm{IR} - 1.27)$.

**Table IV** Equation obtained by principal component analysis

| *Factors* | *C1* |
|---|---|
| (Hb) Anaemia | 0.44347 |
| (I) Renal insufficiency | −0.38982 |
| (PS) Performance status | 0.35096 |
| (PI) Paraprotein index | 0.29178 |

$\mathrm{Score} = \mathrm{Hb} \times 0.44347 - \mathrm{RI} \times 0.38982 + \mathrm{PS} \times 0.35096 + \mathrm{PI} \times 0.29178$.

**Table V** Distribution of patients with unfavorable prognostic factors in the three group categories proposed

| *Factors* | *Clinical stage (%)* A | B | C | *P* |
|---|---|---|---|---|
| Anaemia | | | | |
| ≤8.5 | 0.0 | 20.7 | 66.2 | 0.00005 |
| >8.5 | 100.0 | 79.3 | 33.8 | |
| % Plasma cells | | | | |
| ≤40% | 87.7 | 42.7 | 36.8 | 0.00005 |
| >40% | 12.3 | 57.3 | 63.2 | |
| Urea | | | | |
| ≤40 mg dl$^{-1}$ | 66.7 | 29.1 | 7.7 | 0.00005 |
| >40 mg dl$^{-1}$ | 33.3 | 70.9 | 92.3 | |
| Renal insufficiency | | | | |
| No | 100.0 | 92.7 | 23.5 | 0.00005 |
| Yes | 0.0 | 7.3 | 76.5 | |
| Serum calcium | | | | |
| ≤10 mg dl$^{-1}$ | 64.3 | 72.0 | 51.5 | 0.03 |
| >10 mg dl$^{-1}$ | 35.7 | 28.0 | 48.5 | |
| Infections | | | | |
| Yes | 30.1 | 24.4 | 38.2 | 0.18 |
| No | 61.8 | 75.6 | 69.8 | |
| Bence–Jones proteinuria | | | | |
| Yes | 50.0 | 52.6 | 51.5 | 0.95 |
| No | 50.0 | 47.4 | 48.5 | |
| Performance status | | | | |
| ≤70 | 38.4 | 78.0 | 85.3 | 0.00005 |
| >70 | 61.6 | 22.0 | 14.7 | |
| Paraprotein index | | | | |
| ≤0.09 | 26.0 | 46.3 | 66.2 | 0.00005 |
| >0.09 | 74.0 | 53.7 | 33.8 | |

**Figure 1** Staging system of Group Castellano Leones, classification 1. A, —— 41 months; B, --- 24 months; C, -·-·- 12 months. A versus B versus C, $P < 0.01$.

**Figure 2** Staging system of Durie & Salmon (1975). Stage I, —— 56 months; stage II, --- 26 months; stage III, -·-·- 21 months. I versus II and III, $P < 0.03$; II versus III, n.s.

Carbone *et al.*, 1967; Medical Research Council, 1980), we observed that only the latter permits separation of significantly different patient populations according to survival (Figures 2–5). The survival curves obtained with the MRC staging system were very similar to those obtained with our regression model, although in the model we propose distribution of the patients into the three stages was more uniform and the proportion of wrongly classified patients was lower (35% in the MRC model versus 24% in our model). Moreover, a simplified risk group assignment (Table VI) provided very similar results (Figure 6).

### Discussion

Multiple myeloma is one of the diseases in which prognostic factors have been most extensively investigated (Adachi *et al.*, 1982; Bladé & Rozman, 1984; Brian *et al.*, 1980;

**Figure 3** Staging system of Merlini *et al.* (1980). Stage I, —— 30 months; stage II, --- 23 months; stage III -·-·- 19 months. I versus III, $P < 0.05$; I versus II and II versus III, n.s.

**Figure 5** Staging system of the MRC (1980). Stage A, —— 46 months; stage B, --- 23 months; stage C, -·-·- 13 months. A versus B versus C, $P < 0.01$.

**Figure 4** Staging system of Carbone *et al.* (1967). 0 risk factor, —— 30 months; 1 risk factor, --- 28 months; 2 risk factor, -·-·- 15 months; 3 risk factor, —— 12 months. 0 and 1 versus 2 and 3, $P < 0.0001$.

**Figure 6** Staging system of Group Castellano Leones, classification 2. Stage A, —— 46 months; stage B, --- 23 months: stage C, -·-·- 13 months. A versus B versus C, $P < 0.01$.

**Table VI** Criteria for a simplified risk group assignment

| *Unfavourable characteristics* | *Risk group* |
|---|---|
| Anaemia $\leqslant 8.5\,g\,dl^{-1}$ | A No unfavourable characteristics |
| Renal insufficiency (cr. $>2\,mg\,dl^{-1}$) | B 1 or 2 unfavourable characteristics |
| Performance status $\leqslant 70\%$ | C 3 or 4 unfavourable characteristics |
| Paraprotein index $\leqslant 0.09$ | |

Carbone *et al.*, 1967; Costanzi *et al.*, 1985; Durie & Salmon, 1975; Durie *et al.*, 1980; Hansen & Galton, 1985; Hernández & San Miguel, 1984; Matzner *et al.*, 1978); this has led to the description of numerous staging systems (Alexanian *et al.*, 1975; Costa *et al.*, 1973; Durie & Salmon, 1975; Medical Research Council's Working Party on Leukaemia in Adults, 1980; Merlini *et al.*, 1980; Southeastern Cancer Study Group, 1975). However, few critical papers have been devoted to such classifications (Bettini *et al.*, 1983; Cavagnaro *et al.*, 1980; Gassmann *et al.*, 1985; Pennec *et al.*, 1983; Vercelli *et al.*, 1981) and in fact the model of Durie & Salmon (1975) continues to be the most popular one despite several studies having thrown doubt on its real prognostic value (Bladé & Rozman, 1984; Durie *et al.*, 1980; Gavagnaro *et al.*, 1980; Hernández & San Miguel, 1984; Merlini *et al.*, 1980; Vercelli *et al.*, 1981). In the present work, apart from analysing four of the most popular staging systems, a new model is proposed that was obtained by statistical analysis of principal components; this model also includes a new parameter that relates the monoclonal component with the percentage of plasma cells (paraprotein index).

Univariate analysis of our patients pointed to the existence of nine prognostic factors: poor performance status, infections before diagnosis, renal insufficiency, hypercalcaemia, severe anaemia, Bence-Jones proteinuria, failure to achieve complete remission, high plasma cell infiltration and a low paraprotein index. Recently, Hansen & Galton (1985) have reviewed the prognostic factors with significance for survival in myelomatosis; of them the only important one that does not appear in our study is serum albumin concentration (Alexanian *et al.*, 1975; Bataille *et al.*, 1979). In our series, although hypoalbuminaemia ($<3\,g\,dl^{-1}$) was associated with a shorter survival the differences were not statistically significant. On the other hand, infections before diagnosis, a feature which has not received much attention, was associated with an adverse prognosis in our patients.

Recently, some new parameters, such as thymidine kinase (Simonsson *et al.*, 1985), T-cell subsets (San Miguel *et al.*, 1985), plasma cell morphology (Greipp *et al.*, 1985), plasma cell antigens (Ruiz Arguelles *et al.*, 1984; San Miguel *et al.*, 1987), plasma cell labelling index (Durie *et al.*, 1980) and beta-2-microglobulin (Bataille *et al.*, 1984; Brenning *et al.*, 1986; Cuzick *et al.*, 1985; Van Dobbenburgh *et al.*, 1985) have emerged as possible prognostic factors in multiple myeloma. Of all these, the latter has been the most extensively analysed, being assessed in most studies as an important prognostic variable (Bataille *et al.*, 1984; Brenning *et al.*, 1986; Cuzick *et al.*, 1985). Moreover, in a recent series Cuzick *et al.* (1985) have shown that beta-2- microglobulin was the most powerful independent prognostic factor. We have studied this parameter in 58 MM patients and 21 patients with essential monoclonal gammopathy (data not shown), and have found that the levels beta-2-microglobulin correlated with both the amount of paraprotein and advanced clinical stages and, in addition, could be a useful parameter for the differential diagnosis of monoclonal gammopathies (Ortega *et al.*, 1986). These data show that any analysis of prognostic factors in multiple myeloma should in future include these new variables in order to perfect the prognostic models that will permit a better stratification of the patients and the evaluation of treatment protocols.

In MM patients the M-component synthesized is proportional to the total plasma cell mass (Salmon & Smith, 1970), but neither the monoclonal component nor the percentage of plasma cells, in spite of being key criteria for the diagnosis of MM, have a definite prognostic value (Hansen & Galton, 1985). Accordingly, we thought it of interest to search for a variable of easy clinical application that would relate the production of Igs and plasma cells: the paraprotein index (PI). In our series the patients with a low PI had unfavourable clinico-biological features and shorter survivals. The low capacity of Ig synthesis of these cases could reflect the existence of a blockage in Ig secretion (Qian *et al.*, 1984), and it would be important to rule out that these plasma cells might be at an earlier stage of maturation that could be associated with a different response to chemotherapy (San Miguel *et al.*, 1987).

Although multivariate analyses are much scarcer (Bladé & Rozman, 1984; Cohen *et al.*, 1979; Durie & Salmon, 1975; Durie *et al.*, 1980; Hernández & San Miguel, 1984; Kyle, 1983, 1984; Matzner *et al.*, 1978; Merlini *et al.*, 1980), they have the advantage of considering the simultaneous effect of the different variables on survival, which facilitates the grouping of patients according to the real major prognostic features. The method most commonly employed is the stepwise proportional hazards regression model of Cox (Cox, 1972; Cox & Dakes, 1985). Using this method in our series the best combination of independent prognostic variables in predicting survival was renal function and performance status. These two factors, together with haemoglobin and to a lesser extent calcium, have been those identified with the highest frequency as a significant risk factor in other multivariate analyses (Hansen & Galton, 1985; Harley *et al.*, 1979; Matzner *et al.*, 1978; Merlini *et al.*, 1980).

Using principal component analysis, a statistical approach that has only been used occasionally, we found that the combination of clinical performance status, serum creatinine, haemoglobin and paraprotein index allowed us to discriminate three groups of patients with different survivals. This staging system provided very similar results to those of the MRC (1980), with an even lower level of wrongly classified patients. This is understandable since both models contain similar criteria, with the exception of the paraprotein index which hitherto has not been explored. In contrast, and as evidenced by other studies (Bettini *et al.*, 1983; Bladé & Rozman, 1984; Durie *et al.*, 1980; Gavagnaro *et al.*, 1980; Gassmann *et al.*, 1985; Hansen & Galton, 1985; Hernández & San Miguel, 1984; Merlini *et al.*, 1980; Vercelli *et al.*, 1981), the staging systems proposed by Merlini *et al.* (1980), Durie & Salmon (1975) and Carbone *et al.* (1967) did not discriminate prognostic subgroups.

Our study thus indicates that principal component analysis may be a useful complementary tool for classifying patients according to prognostic factors and the paraprotein index could be a simple parameter of great use in clinical practice.

This work was partially supported by a grant from the Educational Council of Castilla-Leon (Spain) and the Spanish Cancer Association.

### References

ADACHI, T., ASANSO, K., SEZA, K.T., TAKAHASI, I. & KIMURA, I. (1982). Prognostic factors in multiple myeloma treated with prednisolone and sequential melphalan and ifosfamide: MIP combination chemotherapy. *Acta Med. Okuyama.*, **36**, 39.

ALEXANIAN, R., BALCERZAK, S., BONNET, J.D. & 4 others (1975). Prognostic factors in multiple myeloma. *Cancer*, **36**, 1192.

ALEXANIAN, R., BARLOGIE, B. & FRITSCHE, H. (1985). Beta-2-microglobulin in multiple myeloma. *Am. J. Hematol.*, **20**, 345.

BARTL, R., FRISCH, B., BURKHARDT, R. *et al.* (1982). Bone marrow histology in myeloma: Its importance in diagnosis, prognosis, classification and staging. *Br. J. Haematol.*, **51**, 361.

BATAILLE, R., DONADIO, D., MORLOCK *et al.* (1979). Etude retrospective des facteurs pronostique a partir d'une serie de 243 malades. *Rev. Rheum. Malade Osteartic.*, **46**, 77.

BATAILLE, R., GRENIER, J. & SONY, J. (1984). Beta-2-microglobulin in myeloma: Optional use for staging prognosis, and treatment. A prospective study of 160 patients. *Blood*, **63**, 468.

BERGSAGEL, D.E., BAILEY, A.J., LANGLEY, G.R., MACDONALD, R.N., WHITE, D.F. & MILLER, A.B. (1979). The chemotherapy of plasma-cell myeloma and incidence of acute leukemia. *N. Engl. J. Med.*, **301**, 713.

BETTINI, R., STEIDL, L., RAPAZZINI, P. & GIARDINA, G. (1983). Prognostic value of the staging system proposed by Merlini, Waldenström and Jayakar for multiple myeloma. *Acta Haematol.*, **70**, 379.

BLADÉ, J. & ROZMAN, C. (1981). Mieloma múltiple. Análisis de los factores pronóstico de la clasificación por estadios. *Sangre*, **29**, 918.

BRENNING, G., SIMONSSON, B., KLLANDER, C. & AHRE, A. (1986). Pretreatment serum beta-2-microglobulin in multiple myeloma. *Br. J. Haematol.*, **62**, 85.

BRIAN, G.M., DURIE SYDNEY, E., SALMON, S.E. & MOON, T.E. (1980). Pretreatment tumor mass, cell kinetic, and prognosis in multiple myeloma. *Blood*, **55**, 364.

BUCKMAN, R., CUZICK, J. & GALTON, D.A.G. (1982). Long-term survival in myelomatosis. *Br. J. Haematol.*, **52**, 589.

CARBONE, P.P., KELLERHOUSE, L.E. & GEHAN, E.A. (1967). Plasmacytic myeloma. A study of the relationship of survival to various clinical manifestations and anomalus protein type in 112 patients. *Am. J. Med.*, **42**, 937.

COHEN, H.J., SILBERMAN, H.K., LARSEN, W.E., JOHNSON, L. & BARTOLUCCI, A.A. (1979). Combination chemotherapy with intermittent 1-3bis(2-chloroethyl) 1-nitrosurea (BCNU) cyclophosphamide and prednisone for multiple myeloma. *Blood*, **54**, 824.

COMMITTEE OF CHRONIC LEUKEMIA-MYELOMA TASK FORCE, NATIONAL CANCER INSTITUTE (1973). Proposed guidelines protocol studies. II. Plasma cell myeloma. *Cancer Chemother. Rep.*, **4**, 145.

COSTA, G., ENGLE, R.I., JR., SCHILLING, A. & 4 others (1973). Melphalan and prednisone: An effective combination for the treatment of multiple myeloma. *Am. J. Med.*, **54**, 589.

COSTANZI, J.J., COOPER, M.R., SCARFFE, J.H. *et al.* (1985). Phase II study of recombinant alpha-2 interferon in resistant multiple myeloma. *J. Clin. Oncol.*, **3**, 654.

COX, D.R. (1972). Regression models and life tables. *J. Stat. Soc.*, **34**, 187.

COX, D.R. & DAKES, D. (1985). *Analysis of Survival Data, 2nd edn.* Chapman and Hall: London.

CUZICK, J., COOPER, E.H. & MACLENNAN, M. (1985). The prognostic value of serum beta-2-microglobulin compared with either presentation features in myelomatosis. *Br. J. Cancer.*, **52**, 1.

DIXON, W.I. (1985). *BMDP Statistical Software.* University of California Press, Berkeley, CA.

DURIE, B.G.M. & SALMON, S.E. (1975). A clinical staging system for multiple myeloma. Correlation of measured myeloma cell mass with presenting clinical features, response to treatment and survival. *Cancer*, **36**, 844.

DURIE, B.G.M. & SALMON, S.E. (1982). The current status and future prospects of treatment for multiple myeloma. *Clin. Haematol.*, **11**, 181.

DURIE, B.G.M., SALMON, S.E. & MOON, T.E. (1980). Pretreatment tumor mass, cell kinetics and prognosis in multiple myeloma. *Blood*, **55**, 364.

GASSMANN, W., PRALLE, H., HAFERLACH, T. & 4 others (1985). Staging systems for multiple myeloma: A comparison. *Br. J. Haematol.*, **59**, 703.

GAVAGNARO, F., LEIN, J.M., PAVLOVSKY, S. *et al.* (1980). Comparison of two combination chemotherapy regimens for multiple myeloma: Methyl-CCNU, cyclophosphamide and prednisone versus melphan and prednisone. *Cancer Treat. Rep.*, **64**, 73.

GREIPP, P.R., RAYMOND, N.M., KYLE, R.A. & O'FALLON, W.M. (1985). Multiple myeloma: Significance of plasmoblastic subtype in morphological classification. *Blood*, **65**, 305.

HANSEN, O.P. & GALTON, D.A.G. (1985). Classification and prognosis variables in myelomatosis. *Scand. J. Haematol.*, **35**, 10.

HANSEN, O.P., JENSEN, B. & VIDEBACK, A. (1973). Prognosis of myelomatosis on treatment with prednisone and cytostatics. *Scand. J. Haematol.*, **10**, 282.

HARLEY, B., PAJAK, F.P., MCINTYRE, O.R. & 4 others (1979). Improved survival of increased-risk myeloma patients on combined triple-alkylating-agent therapy: A study of CALGB. *Blood*, **54**, 13.

HERNÁNDEZ, J.M. & SAN MIGUEL, J.F. (1984). Factores de pronóstico y estadios en mieloma múltiple. *Sangre*, **29**, 927.

KAPLAN, E.L. & MEIER, P. (1958). Nonparametric estimation from incomplete observations. *J. Am. Stat. Assoc.*, **53**, 457.

KYLE, R.A. (1983). Long-term survival in multiple myeloma. *N. Engl. J. Med.*, **308**, 314.

KYLE, R.A. (1984). Treatment of multiple myeloma. A small step forward? *N. Engl. J. Med.*, **310**, 1382.

MATZNER, Y., BENBASSAT, J. & POLLIACK, A. (1978). Prognostic factors in multiple myeloma. A retrospective study using conventional methods and computer program. *Acta Haematol.*, **60**, 257.

MEDICAL RESEARCH COUNCIL'S WORKING PARTY ON LEUKAEMIA IN ADULTS (1980). Prognostic features in the third MRC myelomatosis trial. *Br. J. Cancer.*, **42**, 831.

MERLINI, G., WALDENSTRÖM, J.G. & JAYAKAR, S.D. (1980). A new improved clinical staging system for multiple myeloma based on analysis of 123 treated patients. *Blood*, **55**, 1011.

ORTEGA, F., CABALLERO, M.D., GARCÍA, J.R. & 4 others (1986). Beta-2-microglobulina en gammapatias monoclonales. *Ann. Med. Intern.* (*Spain*), **5**, 215.

PENNEC, Y., MOTTIER, D., YOUINOV, P. *et al.* (1983). Critical study of staging in multiple myeloma. *Scand. J. Haematol.*, **30**, 182.

PESCE, A., CASSUTO, J.P., GRISOT, C. *et al.* (1983). Etude comparée de deux classifications prognostiques du myélome et recherche d'une corrélation entre plasmocytose médullaire initiale et le pronostic. *Nouv. Rev. Fr. Haematol.*, **25**, 311.

QIAN, G.X., FU, S.M., SOLANKI, D.L. & RAI, K.R.E. (1984). Circulating monoclonal IgM proteins in B cell chronic lymphocytic leukemia: Their identification, characterization and relationship to membrane Ig. *J. Immunol.*, **133**, 3396.

RUIZ-ARGUELLES, G.J., KATZMAN, J.A., GREIPP, P.R., GONCHOROFF, N.J., GARTON, J.P. & KYLE, R.A. (1984). Multiple myeloma: Circulating lymphocytes that express plasma cell antigens. *Blood*, **64**, 352.

SALMON, S.E. & SMITH, B.A. (1970). Immunoglobulin synthesis and total body tumor cell number in IgG multiple myeloma. *J. Clin. Invest.*, **49**, 1119.

SAN MIGUEL, J.F., CABALLERO, M.D. & GONZÁLEZ, M. (1985). T-cell subpopulations in patients with monoclonal gammopathies: Essential monoclonal gammopathy. *Am. J. Haematol.*, **16**, **000**.

SAN MIGUEL, J.F., MORO, M. & GONZÁLEZ, M. (1987). Plasmoblastic multiple myeloma: An immunological different subtype. *Br. J. Haematol.*, **66**, 275.

SIMONSSON, B., KOLLANDER, C.F., BRENNING, G., KILLANDER, A., AHNE, A. & GRONOWITZ, J.S. (1985). Evaluation of serum seosythymidine kinase as a marker in multiple myeloma. *Br. J. Haematol.*, **61**, 215.

VAN DOBBENBURGH, O.A., RODENHUIS, S., OCKVIZEN, T.H. *et al.* (1985). Serum beta-2-microglobulin, a real improvement in the management of multiple myeloma? *Br. J. Haematol.*, **61**, 611.

VERCELLI, D., COZZALMO, F. & DIGUGLIELMO, R. (1981). A comparison of two staging system for multiple myeloma. *Nouv. Rev. Fr. Haematol.*, **23**, 107.

VERCELLI, D., DIGUGLIELMO, R., GUIDI, G., SCOLARI, L., BURICCHI, L. & COZZOLINO, F. (1980). Bone marrow percentages of plasma cells in the staging of monoclonal gammopathies. *Nouv. Rev. Fr. Haematol.*, **22**, 139.